# Predictive cytogenetic biomarkers for colorectal neoplasia in medium risk patients

**Published:** 2015

**Authors:** EM Ionescu, T Nicolaie, MA Ionescu, G Becheanu, F Andrei, M Diculescu, M Ciocirlan

**Affiliations:** * “Carol Davila” University of Medicine and Pharmacy, Bucharest, Romania; ** Genetic Lab, Bucharest, Romania

**Keywords:** colorectal cancer, screening, biomarkers, cytokinesis, nuclear division index

## Abstract

**Rationale:** DNA damage and chromosomal alterations in peripheral lymphocytes parallels DNA mutations in tumor tissues.

**Objective:** The aim of our study was to predict the presence of neoplastic colorectal lesions by specific biomarkers in “medium risk” individuals (age 50 to 75, with no personal or family of any colorectal neoplasia).

**Methods and Results:** We designed a prospective cohort observational study including patients undergoing diagnostic or opportunistic screening colonoscopy. Specific biomarkers were analyzed for each patient in peripheral lymphocytes - presence of micronuclei (MN), nucleoplasmic bridges (NPB) and the Nuclear Division Index (NDI) by the cytokinesis-blocked micronucleus assay (CBMN). Of 98 patients included, 57 were “medium risk” individuals. MN frequency and NPB presence were not significantly different in patients with neoplastic lesions compared to controls. In “medium risk” individuals, mean NDI was significantly lower for patients with any neoplastic lesions (adenomas and adenocarcinomas, AUROC 0.668, p 00.5), for patients with advanced neoplasia (advanced adenoma and adenocarcinoma, AUROC 0.636 p 0.029) as well as for patients with adenocarcinoma (AUROC 0.650, p 0.048), for each comparison with the rest of the population. For a cut-off of 1.8, in “medium risk” individuals, an NDI inferior to that value may predict any neoplastic lesion with a sensitivity of 97.7%, an advanced neoplastic lesion with a sensitivity of 97% and adenocarcinoma with a sensitivity of 94.4%.

**Discussion:** NDI score may have a role as a colorectal cancer-screening test in “medium risk” individuals.

**Abbreviations:** DNA = deoxyribonucleic acid; CRC = colorectal cancer; EU = European Union; WHO = World Health Organization; FOBT = fecal occult blood test; CBMN = cytokinesis-blocked micronucleus assay; MN = micronuclei; NPB = nucleoplasmic bridges; NDI = Nuclear Division Index; FAP = familial adenomatous polyposis; HNPCC = hereditary non-polypoid colorectal cancer; IBD = inflammatory bowel diseases; ROC = receiver operating characteristics; AUROC = area under the receiver operating characteristics curve.

## Introduction

Colorectal cancer (CRC) is a major public health issue [**1**-**3**]. Based on demographic trends and on incidence and mortality rates, predictive models estimate by 2030 an alarming increase of CRC incidence and mortality up to 80% and 50% respectively [**1**].

CRC is the most common newly diagnosed cancer and the second most common cause of cancer related death in Europe [**4**]. Romania has one of the highest CRC incidence rate in Europe - 43.9/ 100000 cases in women and 88.6/ 100000 in men, with a mortality rate of 20.2/ 100000 in women and 46.9/ 100000 in men. These figures have increased twofold in the last 20 years [**4**].

75% of CRC cases are sporadic and more than 90% of CRC arise in individuals over the age of 50. The 5-year survival rate is of 90% if CRC is diagnosed when localized to the bowel wall, no more than 68% if regional lymph node metastases are present and only 10% if there are distant metastases [**5**]. Symptoms usually appear in locally advanced or metastatic stages of the disease, mostly lower gastrointestinal bleeding, iron deficiency anemia, changes in bowel habit, abdominal pain and weight loss.

One way of improving these numbers is to detect and treat the disease in its asymptomatic premalignant stages by screening individuals at risk. A simple, highly sensitive “screening test” is used to select individuals from a population at risk and cases with positive tests will be subsequently checked with a “confirmation test”. CRC is particularly suitable for this as it complies with a set of “screening principles” defined in 1968 by the World Health Organization (WHO) [**6**]. It has a high prevalence, it arises from precursor lesions (adenomas) according to a long adenoma-carcinoma sequence and, most importantly, curative excision of adenomas in this interval prevents CRC development [**7**]. 

Screening is recommended for asymptomatic individuals aged between 50 and 75, without personal or family history of adenoma or adenocarcinoma (“the medium risk group”). Screening of these individuals reduces CRC incidence and mortality by detecting and removing significant adenomas and detecting cancers in early curable stages [**7**-**12**]. Currently, only 12 EU member states have implemented population-based CRC screening programs as recommended in 2003 by the European Council [**13**,**14**]. Romania has not such a program yet.

Currently used CRC “screening tests” are: fecal occult blood testing (FOBT) in France or flexible sigmoidoscopy in United Kingdom [**15**,**16**], with colonoscopy as “confirmation test”. There is no “gold standard”, as colonoscopy may have false negative results. Colonoscopy maybe be directly used as “screening and confirmatory test” and this approach is increasingly preferred in Europe (Poland or Germany) and United States [**17**-**19**]. Screening colonoscopy has decreased CRC incidence and mortality by 65% as compared to non-screened population [**20**]. However, it is expensive and carries certain risks such as post polypectomy hemorrhages or perforations (more frequently in elders or in associated diverticular disease) as well as sedation related complications.

Can we have better screening tests than the actual ones? We know that DNA damage and chromosomal alterations in peripheral lymphocytes parallels with DNA mutations in tumor tissues [**21**]. Our previous work pointed out the role of certain cytogenetic biomarkers in predicting neoplastic colorectal lesions [**22**,**23**]. We quantified the peripheral lymphocytes DNA damage by the cytokinesis-blocked micronucleus assay (CBMN) method. Through this technique, three specific biomarkers in peripheral lymphocytes were assessed – the presence of micronuclei (MN), of nucleoplasmic bridges (NPB) and the nuclear division index (NDI). We proved that these cytogenetic markers have a certain predictive value for CRC adenoma and adenocarcinoma presence and maybe candidates for “screening tests”.

NPB presence is a direct proof of genome alteration by DNA repairing defects or telomere fusion. We have proved that, in general population, NPB were significantly less frequent in patients with advanced adenomas or CRC when compared with patients with normal colonoscopy, hyperplastic polyps or non-advanced adenomas [**22**].

NDI is a marker of cell proliferation in cultures and is considered a measure of general toxicity. Cells with greater chromosomal damage will either die before cell division or may be less able to enter in this phase [**24**-**27**]. Therefore, more a cell will accumulate genetic alterations, less it will be able to divide and NDI will be lower. We have proved that, in the general population, mean NDI was significantly lower in patients with neoplastic lesions (CRC and adenomas) than in patients with normal colonoscopy [**23**].

The aim of our study was to assess the MN, NPB and NBI predictive role of neoplastic colorectal lesions in “medium risk group” individuals. 

## Materials and Methods

Based on our previous experience [**23**], since 2011, we have designed a prospective cohort observational study of patients who have undergone colonoscopies and cytogenetic testing. 

Cytogenetic testing was proposed in our department to all consecutive patients with CRC neoplastic lesions (hyperplastic polyps, adenomas, adenocarcinomas) or with normal colonoscopy in a 1:1 ratio. The indications of colonoscopy were both for “diagnostic purposes” in symptomatic patients or for “opportunistic screening” for asymptomatic patients. Patients had to be above 18 and signed an inform consent.

We excluded patients with personal history of familial adenomatous polyposis (FAP) or hereditary non-polypoid colorectal cancer (HNPCC), patients with inflammatory bowel diseases (IBD) or other acute or chronic colitis, history of malignancy or radiation exposure.

We performed a subgroup analysis of our prospective database by selecting and analyzing patients belonging to the “medium risk group” (age between 50 and 75, without personal or family history of adenoma or adenocarcinoma). The local ethical committee approved the study protocol.

When 2 or more lesions were synchronously present, the more advanced neoplastic lesion was retained as the “outcome of colonoscopy”. Adenomas were classified according to WHO criteria: tubular, villous and tubular-villous [**28**]. Dysplasia was graded by Vienna classification [**29**]. Advanced adenomas have ≥10 mm size, high-grade dysplasia or have a minimum 25% of villous component [**30**].

We obtained a blood sample form each patient to be analyzed by the CBMN technique as previously described [**23**,**24**]. We used the definition criteria from Fenech et al. [**31**] for scoring MN and NPB. NDI was calculated according to Eastmond, Tucker et al. [**32**]: NDI = (M1 +2M2 +3M3 +4M4)/N, where M1, M2, M3 and M4 indicate the number of cells with one, two, three and four nuclei and N the total number of cells analyzed (N = 500). 

For statistical analysis, we used the SPSS 16.0 software. Quantitative variables were expressed as means and ranges. Categorical variables were presented as so and as percentages. Predictive ROC curves for dichotomous outcome categorical variables were constructed for measured quantitative variables. Sensitivities and specificities were explored for different cut-off values on the ROC curve. A p value of less than 0.05 was considered statistically significant.

## Results

From a database population of 98 patients, we selected 57 at “medium risk” for CRC. Demographic characteristics of patients and colonoscopy findings are summarized in **Table 1**. 

**Table 1:** The demographic characteristics of patients and colonoscopy findings

	Medium risk group	All patients
**Patients** (n)	57	98
**Women** (n,%)	28 (49.1%)	50 (51%)
**Smoking** (n, %)	9 (15.8%)	21 (21.4%)
**Age** (mean, range)	60.57 (51-75)	55.36 (24-75)
Personal history		
• no history (n,%)	57 (100%)	84 (85.7%)
• adenoma (n,%)	0	9 (9.2%)
• adenocarcinoma (n,%)	0	5 (5.1%)
Family history		
• no history (n,%)	57 (100%)	86 (87.8%)
• adenoma (n,%)	0	2 (2%)
• adenocarcinoma (n,%)	0	10 (10.2%)
Colonoscopy		
• normal (n,%)	22 (38.6%)	48 (49%)
• hyperplastic polyps (n,%)	2 (3.5%)	6 (6.1%)
• adenoma (n,%)	19 (33.3%)	26 (26.5%)
[of which advanced] (n,%)	[12 (21.1%)]	[15 (15.3%)]
• adenocarcinoma (n,%)	15 (26.3%)	18 (18.4%)

The percentage of normal colonoscopies in the “medium risk group” was 38.6%. As the inclusion ratio of abnormal to normal colonoscopy was 1:1, the percentage of normal colonoscopies in all patients was close to 50%.

MN and NPB frequencies were not significantly different in patients with neoplastic lesions versus patients with normal colonoscopy, both in the “medium risk group” and in the whole group. 

**Fig. 1:**
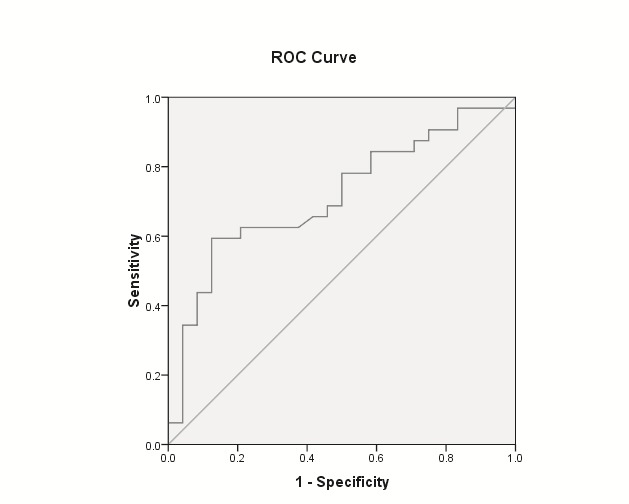
AUC ROC of NDI to predict any colorectal neoplasia (adenoma and adenocarcinoma) for “medium risk group” individuals

**Fig. 2:**
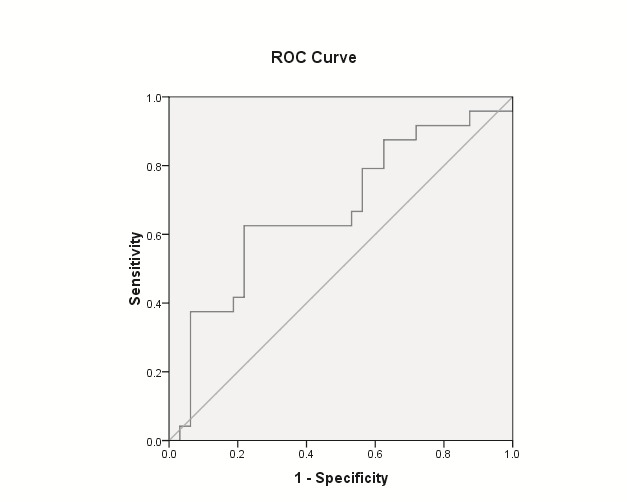
AUC ROC of NDI to predict colorectal advanced neoplasia (advanced adenoma and cancer) for “medium risk group” individuals

**Fig. 3:**
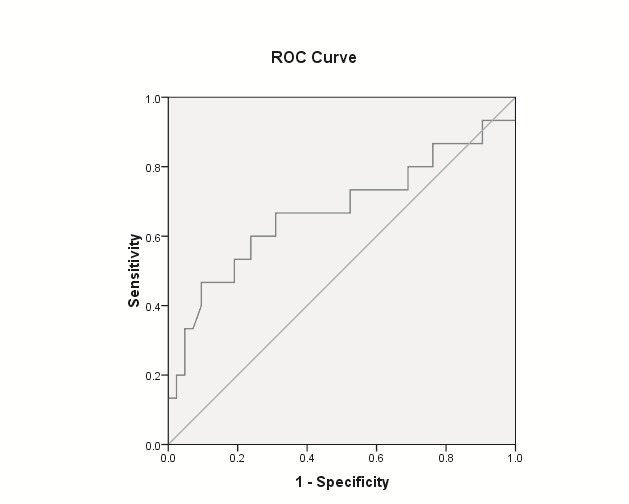
AUC ROC of NDI to predict colorectal adenocarcinoma for “medium risk group” individuals

Mean NDI was significantly lower for patients with any neoplastic lesions (adenomas and adenocarcinomas), for patients with advanced neoplasia (advanced adenoma and adenocarcinoma) as well as for patients with adenocarcinoma, for each comparison with the rest of the population. The findings were for the “medium risk group” as well as for the whole group (**Table 2** and **Fig. 1**-**3**). 

**Table 2:** The predictive value of NDI in colorectal neoplastic lesions - any neoplasia, advanced neoplasia and adenocarcinoma

	Medium risk group	All patients
**Any neoplasia** (adenoma & adenocarcinoma)	AUROC = 0.715	AUROC = 0.668
	P = 0.006 (**Fig. 1**)	P = 0.005
	NDI < cut-off 1.8	NDI < cut-off 1.8
	sensitivity = 96.9%	sensitivity = 97.7%
	specificity = 16.7%	specificity = 20.4%
**Advanced neoplasia** (advanced adenoma & adenocarcinoma)	AUROC = 0.672	AUROC = 0.636
	P = 0.029 (**Fig. 2**)	P = 0.029
	NDI < cut-off 1.8	NDI < cut-off 1.8
	sensitivity = 95.8%	sensitivity = 97%
	specificity = 12.5%	specificity = 17.2%
Adenocarcinoma	AUROC = 0.672	AUROC = 0.650
	P = 0.049 (**Fig. 3**)	P = 0.048
	NDI < cut-off 1.8	NDI < cut-off 1.8
	sensitivity = 93.3%	sensitivity = 94.4%
	specificity = 9.5%	specificity = 13.8%

## Discussion

The CBMN technique is one method of assessing chromosomal alterations induced by occupational or environmental factors [**33**-**36**]. CBMN allows the identification of specific predictive biomarkers of different neoplasias [**37**-**42**]. It may reflect changes in mitotic division, apoptosis, necrosis, chromosomal loss or deletions. Frequency of mononuclear cells in cultures provides valuable information about the level of chromosomal or genomic mutations that accumulate in vivo [**31**,**43**,**44**].

We excluded individuals with prior exposure to ionized radiation (accidentally exposed or for medical and occupational reasons) and with history of neoplasia, as these are confounding factors in studies addressing changes in neoplastic disease [**21**,**45**-**50**]. Although some published studies showed a gender and age influence of MN frequency and NDI [**51**-**53**], we did not correct results for age and sex as recent studies did not confirm such hypothesis [**54**,**55**]. 

MN frequency was not predictive of significant colorectal neoplastic lesions. This is consistent with what we have previously reported, in a smaller group of patients [**22**,**23**], but in contradiction with the results of Karaman et al. [**56**]. Their team found that MN frequency is significantly increased in patients with adenoma and adenocarcinoma compared with controls (3.72 ± 1.34, 3.58 ± 1.21 vs. 1.97 ± 0.81, p < 0.001). A recent study [**57**] reported a significant higher MN frequency in patients with thyroid cancer (37.58±3.07) versus non-neoplastic thyroid pathology (6.60±1.29, 14.90±1.69, and 15.56± 1.76, p <0.05).

El-Zein et al. [**46**] proposed NDI as a screening marker for lung cancer in smokers. We previously pointed out that NDI is significantly lower in peripheral lymphocytes of patients with any neoplastic epithelial colorectal lesions than in individuals without such lesions [**22**,**23**]. Here, in a larger group of patients, we proved that this statement was still valid for individuals at medium risk for CRC. In patients with neoplastic colorectal lesions, circulating lymphocytes express DNA damages that prevent survival during cellular cycle division. A smaller number of cells are able to complete cellular division in lymphocytic culture in patients with colorectal neoplasia, than in control patients, with similar environmental and occupational risk factors. One explanation is that cells will die before finishing the first division, or will suffer a mitotic delay which, by not allowing the repair of genotoxic lesions, will modify the number of cells entering the mitosis and the proportion of mono/bi-/tri-/ and tetra-nucleated cells. Another explanation is a clastogenic effect of mutagens with an aneugenic action, inducing a blockade of the cell cycle, with less dividing cells and consequently low NDI.

Although molecular biology techniques may test a large panel of DNA, RNA and proteomic molecules, up to now it did not identify reliable biomarkers for CRC screening [**58**]. Also, their costs are significantly higher than cytogenetic techniques. Instead, CBMN can be widely used screening protocols for the detection of cytological alterations in individuals at risk for cancer. A value of NDI of less than 1.8 has high sensitivity for any colorectal neoplasia detection and may be used as a CRC screening test in “medium risk” individuals. Future development of automatic laser scanning CBMN will hopefully improve the technique speed, costs and accuracy [**59**-**61**].

One of the limitations of this paper is that data is based on a subgroup analysis of our patients’ database. However, data was prospectively collected and, as an internal validation checkpoint, it was consistent with our previous finding and over the entire group of patients. Another limitation is that we did not record the colonoscopy indication, diagnostic or opportunistic screening. This may as well be regarded as an advantage as it may be used for both diagnostic and screening purposes. 

**Sources of funding:** This work has been financially supported by the European Social Fond through POSDRU/159/1.5/S/133377 grant program.

**Disclosures:** none

## References

[1] Karsa LV, Lignini TA, Patnick J, Lambert R, Sauvaget C (2010). The dimensions of the CRC problem. Best Practice & Research Clinical Gastroenterology.

[2] Huxley RR, Clifton P (2009). The impact of dietary and lifestyle risk factors on risk of colorectal cancer: a quantitative overview of the epidemiological evidence. Int J Cancer.

[3] (2008). World Cancer Report. Cancer site by site – colorectal cancer. In: Boyle P, Levin B. World cancer report 2008.

[4] Ferlay J, Parkin DM, Steliarova-Foucher E (2010). Estimates of cancer incidence and mortality in Europe in 2008. Eur J Cancer.

[5] Altekruse SF, Kosary CL, Waldron W (1975–2007). SEER Cancer Statistics Review, National Cancer Institute.

[6] Wilson JMG, Jungner YG (1968). Principles and practice of mass screening for disease.

[7] Citarda F, Tomaselli G, Crespi M (2001). Efficacy in standard clinical practice of colonoscopic polypectomy in reducing colorectal cancer incidence. Gut.

[8] Arditi C, Peytremann-Bridevaux I, Burnand B (2009). Appropriateness of colonoscopy in Europe (EPAGE II). Screening for colorectal cancer. Endoscopy.

[9] Bertario L, Russo A, Sala P (2003). Predictors of metachronous colorectal neoplasms in sporadic adenoma patients. Int J Cancer.

[10] Jorgensen OD, Kronborg O, Fenger C, Rasmussen M (2007). Influence of long-term colonoscopic surveillance on incidence of colorectal cancer and death from the disease in patients with precursors (adenomas). Acta Oncol.

[11] Thiis-Evensen E, Hoff GS, Sauar J, Langmark F, Majak BM, Vatn MH (1999). Population-based surveillance by colonoscopy: effect on the incidence of colorectal cancer. Scand J Gastroenterol.

[12] Winawer SJ, Zauber AG, Ho MN, O Brien MJ, Gottlieb LS, Sternberg SS, Waye JD, Schapiro M, Bond JH, Panish JF (1993). Prevention of colorectal cancer by colonoscopic polypectomy (The National Polyp Study Workgroup). N Engl J Med.

[13] Segnan N, Patnick J, von Karsa L (2010). European guidelines for quality assurance in colorectal cancer screening and diagnosis.

[14] (2008). Commission of the European Communities, Report from the commission to the council, the European Parliament, the European Economic and Social committee and the Committee of the Regions - Implementation of the Council Recommendation of 2 December 2003 on cancer screening (2003/878/EC).

[15] Weitz J, Koch M, Debus J, Hohler T (2005). Colorectal cancer. Lancet.

[16] Hayat JM, Howlader N, Reichman ME, Edwards BK (2007). Cancer Statistics, Trends, and Multiple Primary Cancer Analyses from the Surveillance, Epidemiology, and End Results (SEER) Program. The Oncologist.

[17] Rex DK, Bond JH, Winawer S, Levin TR, Burt RW, Johnson DA, Kirk LM, Litlin S, Lieberman DA, Waye JD, Church J, Marshall JB, Riddell RH (2002). Quality in the technical performance of colonoscopy and the continuous quality improvement process for colonoscopy: recommendations of the US Multi- Society Task Force on Colorectal Cancere. Am J Gastroenterol.

[18] Rex DK, Petrini JL, Baron TH, Chak A, Cohen J, Deal SE, Hoffman B, Jacobson BC, Mergener K, Petersen BT, Safdi MA, Faigel DO, Pike IM (2006). Quality indicators for gastrointestinal endoscopic procedures: an introduction. Am J Gastroenterol.

[19] Lieberman D, Nadel M, Smith RA (2007). Standardized colonoscopy reporting and data system: report of the Quality Assurance Task Group of the National Colorectal Cancer Roundtable. Gastrointest Endosc.

[20] Kahi CJ, Imperiale TF, Juliar BE (2009). Effect of screening colonoscopy on colorectal cancer incidence and mortality. Clin Gastroenterol Hepatol.

[21] Iarmarcovai G, Ceppi M, Botta A (2008). Micronuclei frequency in peripheral blood lymphocytes of cancer patients: a meta-analysis. Mutat Res.

[22] Ionescu ME, Ciocirlan M, Ionescu C, Becheanu G, Gologan S, Teiusanu A, Arbanas T, Diculescu MM (2011). Genetic biomarkers for neoplastic colorectal cancer in peripheral lymphocytes. Maedica.

[23] Ionescu ME, Ciocirlan M, Becheanu G, Nicolaie T, Ditescu C, Teiusanu A, Gologan S, Arbanas T, Diculescu M (2011). Nuclear Division Index may predict neoplastic colorectal lesions. Maedica.

[24] Palus J, Rydzynski K, Dziubaltowska E (2003). Genotoxic effects of occupational exposure to lead and cadmium. Mutat. Res.

[25] Santos-Mello R, Kwan D, Norman A (2004). Chromosome aberrations and T-cell survival in human lymphocytes. Radiat. Res.

[26] Nath CJ (1999). Micronuclei assay in cytokinesis-blocked binucleated and conventional mononucleated methods in human peripheral lymphocytes. Teratog. Carcinog. Mutagen.

[27] Minozzo R, Deimling LI, Petrucci Gigante L (2004). Micronuclei in peripheral blood lymphocytes of workers exposed to lead. Mutation Research.

[28] Morson BC, Sobin LH (1976). Histological typing of intestinal tumours. In: International Histological Classification of Tumours, No. 15.

[29] Schlemper RJ, Riddell RH, Kato Y (2000). The Vienna classification of gastrointestinal epithelial neoplasia. Gut.

[30] Winawer SJ, Zauber AG (2002). The advanced adenoma as the primary target of screening. Gastrointest Endosc Clin N Am.

[31] Fenech M, Chang WP, Kirsch-Volders M (2003). HUMN project: detailed description of the scoring criteria for the cytokinesis-block micronucleus assay using isolated human lymphocyte cultures. Mutation Res.

[32] Eastmond DA, Tucker JD (1989). Identification of aneuploidy-inducing agents using cytokinesis-blocked human lymphocytes and an antikinetochore antibody. Environ. Mol. Mutagen.

[33] Fenech M (2000). The in vitro micronucleus technique. Mutat Res.

[34] Fenech M (2010). The lymphocyte cytokinesis-block micronucleus cytome assay and its application in radiation biodosimetry. Health Phys.

[35] Vral A, Fenech M, Thierens H (2011). The micronucleus assay as a biological dosimeter of in vivo ionising radiation exposure. Mutagenesis.

[36] Fenech M (2007). Cytokinesis-block micronucleus cytome assay. Nat. Protoc.

[37] Fenech M (2002). Chromosomal biomarkers of genomic instability relevant to cancer. Drug Discov Today.

[38] Joseph LJ, Raut YS, Kand P, Hawaldar RW, Nair N (2009). Micronuclei frequency in peripheral blood lymphocytes of thyroid cancer patients after radioiodine therapy and its relationship with metastasis. Mutat Res.

[39] Herrmann M (2003). Standard and molecular cytogenetics of endocrine tumors. Am J Clin Pathol.

[40] Gil OM, Oliveira NG, Rodrigues AS, Laires A, Ferreira TC, Limbert E, Rueff J (2000). No evidence of increased chromosomal aberrations and micronuclei in lymphocytes from nonfamilial thyroid cancer patients prior to radiotherapy. Cancer Genet Cytogenet.

[41] El-Zein R, Vral A, Etzel CJ (2011). Cytokinesis-blocked micronucleus assay and cancer risk assessment. Mutagenesis.

[42] El-Zein R, Fenech M, Lopez MS, Spitz MR, Etzel CJ (2008). Cytokinesis-Bloked Micronucleus Assay biomarkers identify lung cancer cases among smokers. Cancer Epidemiol Biomarkers Prev.

[43] Kirsch-Volders M, Fenech M (2001). Inclusion of micronuclei in non-divided mononuclear lymphocytes and necrosis/ apoptosis may provide a more comprehensive cytokinesis block micronucleus assay for biomonitoring purposes. Mutagenesis.

[44] Kirsch-Volders M (1997). The in vitro MN test: a multi-endpoint assay to detect simultaneously mitotic delay, apoptosis, chromosomal breakage, chromosome loss and non disjunction. Mutat Res.

[45] Minicucci EM, Ribeiro DA, de Camargo B (2008). DNA damage in lymphocytes and bucal mucosa cells of children with malignant tumours undergoing chemotherapy. Clin Exp Med.

[46] Murgia E, Ballardin M, Bonassi S (2008). Validation of micronuclei frequency in peripheral blood lymphocytes as early cancer risk biomarker in a nested case-control studyg. Mutation Research/ Fundamental and Molecular Mechanisms of Mutagenesis.

[47] Jianlin L, Jiliang L, Lifen J (2006). Variation of ATM protein expression in response to irradiation of lymphocytes in lung cancer patients and controls. Toxicology.

[48] Lou J, Zheng W (2007). Investigating the genetic instability in the peripheral lymphocytes of 36 untreated lung cancer patients with comet assay and micronucleus assay. Mutat. Res.

[49] Varga D, Hoegel J, Maier C (2006). On the difference of micronucleus frequencies in peripheral blood lymphocytes between breast cancer patients and controls. Mutagenesis.

[50] Baciuchka-Palmaro M, Orsiere T, Duffaud F (2002). Acentromeric micronuclei are increased in peripheral blood lymphocytes of untreated cancer patients. Mutat. Res.

[51] Kazimirova A, Barancokova M, Dzupinkova Z, Wsolova L (2009). Micronuclei and chromosomal aberrations, important markers of ageing: possible association with XPC and XPD polymorphisms. Mutat. Res.

[52] Fenech M, Bonassi S (2011). The effect of age, gender, diet and lifestyle on DNA damage measured using micronucleus frequency in human peripheral blood lymphocytes. Mutagenesis.

[53] Thierens H, Vral AM, Mohier R (2000). Cytogenetic monitoring hospital workers occupationally exposed ionizing radiation using the micronucleus centromere assay. Mutagenesis.

[54] Scarpato R, Verola C, Fabiani B (2011). Nuclear damage in peripheral lymphocytes of obese and overweight Italian children as evaluated by the γ-H2AX focus assay and micronucleus test. FASEB J.

[55] Gövercin M, Tomatır AG, Evyapan F, Acikbas I, Coskun I, Akdag B (2014). Elevated micronucleus frequencies in patients with pleural plaque secondary to environmental exposure to asbestos. Genetics and Molecular Research.

[56] Karaman A, Binici DN, Kabalar ME (2008). Micronucleus analysis in patients with colorectal adenocarcinoma and colorectal polyps. World J Gastroenterol.

[57] Al Faisal AH, Al-Ramahi IJ, Abdul-Hassan I (2014). Micronucleus frequency among Iraqi thyroid disorder patients. Comp Clin Pathol.

[58] Luna Coronell JA, Sergelen K (2012). The current status of cancer biomarker research using tumour-associated antigens for minimal invasive and early cancer diagnostics. J Proteomics.

[59] Decordier IA, Papine G, Plas S, Roesems K, VandeLoock J, Moreno-Palomo E, Cerneli D, Anderson D (2009). Automated image analysis of cytokinesis-blocked micronuclei: an adapted protocol and a validated scoring procedure for biomonitoring. Mutagenesis.

[60] Schunck C, Johannes T, Varga DT, Lorch T, Plesch A (2004). New developments in automated cytogenetic imaging: unattended scoring of dicentric chromosomes, micronuclei, single cell gel electrophoresis, and fluorescence signals. Cytogenet. Genome Res.

[61] Fenech M, Kirsch-Volders M, Rossnerova A, Sram R, Romm H, Bolognesi C, Ramakumar A, Soussaline F (2013). HUMN project initiative and review of validation, quality control and prospects for further development of automated micronucleus assays using image cytometry systems. Int. J. Hyg. Environ. Health.

